# A data-driven metapopulation model for the Belgian COVID-19 epidemic: assessing the impact of lockdown and exit strategies

**DOI:** 10.1186/s12879-021-06092-w

**Published:** 2021-05-30

**Authors:** Pietro Coletti, Pieter Libin, Oana Petrof, Lander Willem, Steven Abrams, Sereina A. Herzog, Christel Faes, Elise Kuylen, James Wambua, Philippe Beutels, Niel Hens

**Affiliations:** 1 Data Science Institute, I-Biostat, Hasselt University, Agoralaan Gebouw D, Diepenbeek, 3590 Belgium; 2 Vrije Universiteit Brussel, Pleinlaan 2, Brussels, 1050 Belgium; 3 Rega Institute for Medical Research, Katholieke Universiteit Leuven, Herestraat 49, Leuven, 3000 Belgium; 4 Centre for Health Economics Research and Modelling Infectious Diseases, Vaccine and Infectious Disease Institute, University of Antwerp, Universiteitsplein 1, Wilrijk, 2610 Belgium; 5 Global Health Institute, Family Medicine and Population Health, University of Antwerp, Wilrijk, Belgium; 6 Institute for Medical Informatics, Statistics and Documentation, Auenbruggerplatz 2, Graz, 8036 Austria; 7 School of Public Health and Community Medicine, The University of New South Wales, Sydney, Australia

**Keywords:** COVID-19, Behavioral changes, Metapopulation, Epidemic modeling, Spatial transmission, Mixing patterns

## Abstract

**Background:**

In response to the ongoing COVID-19 pandemic, several countries adopted measures of social distancing to a different degree. For many countries, after successfully curbing the initial wave, lockdown measures were gradually lifted. In Belgium, such relief started on May 4th with phase 1, followed by several subsequent phases over the next few weeks.

**Methods:**

We analysed the expected impact of relaxing stringent lockdown measures taken according to the phased Belgian exit strategy. We developed a stochastic, data-informed, meta-population model that accounts for mixing and mobility of the age-structured population of Belgium. The model is calibrated to daily hospitalization data and is able to reproduce the outbreak at the national level. We consider different scenarios for relieving the lockdown, quantified in terms of relative reductions in pre-pandemic social mixing and mobility. We validate our assumptions by making comparisons with social contact data collected during and after the lockdown.

**Results:**

Our model is able to successfully describe the initial wave of COVID-19 in Belgium and identifies interactions during leisure/other activities as pivotal in the exit strategy. Indeed, we find a smaller impact of school re-openings as compared to restarting leisure activities and re-openings of work places. We also assess the impact of case isolation of new (suspected) infections, and find that it allows re-establishing relatively more social interactions while still ensuring epidemic control. Scenarios predicting a second wave of hospitalizations were not observed, suggesting that the per-contact probability of infection has changed with respect to the pre-lockdown period.

**Conclusions:**

Contacts during leisure activities are found to be most influential, followed by professional contacts and school contacts, respectively, for an impending second wave of COVID-19. Regular re-assessment of social contacts in the population is therefore crucial to adjust to evolving behavioral changes that can affect epidemic diffusion.

**Supplementary Information:**

The online version contains supplementary material available at (10.1186/s12879-021-06092-w).

## Background

The COVID-19 pandemic has put a massive burden on modern society. While the global death toll of the virus has risen above 500,000 reported deaths on the 15th of July [1], several countries are evaluating strategies to cope with the virus on the medium to long term. As during the first wave of the COVID-19 pandemic neither a vaccine nor adequate therapeutic options were available, non-pharmaceutical interventions have been proven effective in reducing the pressure on healthcare systems [2–6]. After a massive implementation of lockdown measures, affecting as much as one third of the global world population [7], governments have eased some of the social distancing measures. After imposing a lockdown on March 14th [8], the Belgian government curtailed some of these measures with a plan for a gradual reopening over several weeks, starting from the 4th of May. The absence of substantial population immunity after this first wave of COVID-19 in Belgium [9] increases the risk of subsequent large-scale outbreaks when interventions are relaxed which could result, when not contained, in new COVID-19 waves with large numbers of new confirmed cases and hospitalized persons. In this context, data-driven models of disease spread can provide useful insights into the expected impact of easing non-pharmaceutical interventions [2, 6, 10]. Here we present a scenario analysis of possible re-opening strategies easing lockdown measures based on a data-driven metapopulation model for Belgium for COVID-19 [11]. We compare the expected epidemic trajectories and, at the same time, we validate the modelled scenarios with social contact data collected during and after lockdown. We aim to identify which intervention strategies have the largest potential impact on disease spread, based on the scarce data available during the early stage of the pandemic.

## Methods

We constructed a meta-population model for COVID-19, in order to study the Belgian epidemic. The model reproduces the demography of *children* (0-18 years) and *adults* (19 years and above) in the 581 different Belgian municipalities [12]. Publicly available data [13, 14] from a social contact survey conducted in Flanders (Belgium) anno 2010-2011 is used to inform mixing patterns of the population [15–17]. Mobility data retrieved from the Belgian census [18] is used to reconstruct mobility fluxes due to school attendance and work. A stochastic compartmental model is used to describe the spread of COVID-19 in the population within each patch of the system. The model is fitted to national hospitalization data [19].

### Compartmental patch model

**Fig. 1:**
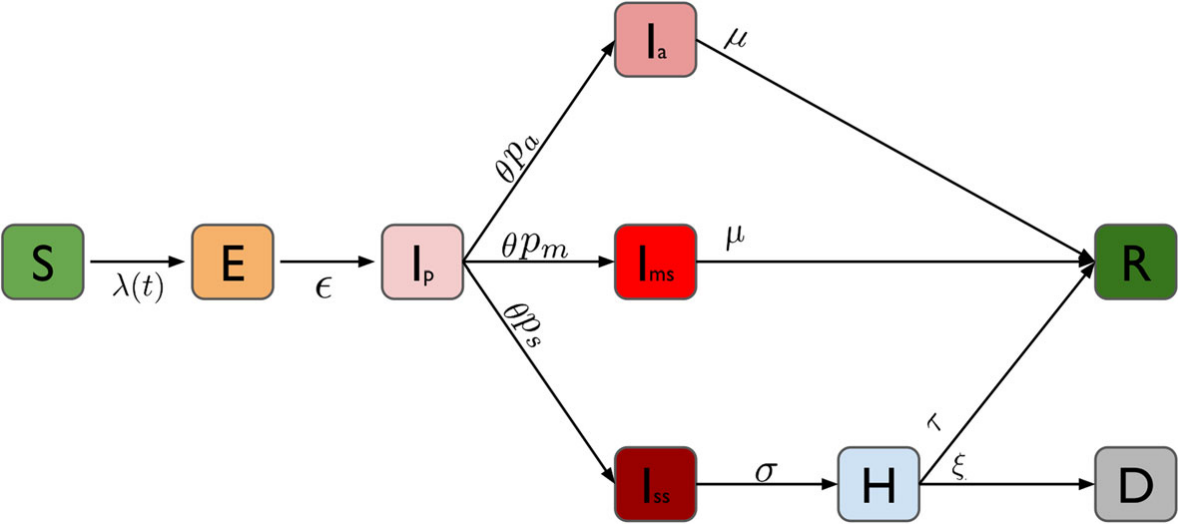
Schematic representation of the compartmental model: Individuals start as susceptible (*S*) and can become exposed to the disease (*E*) when interacting with infected individuals (*I*_*p*_,*I*_*a*_,*I*_ms_ and *I*_ss_). After a latent period, exposed individuals enter a pre-symptomatic phase (*I*_*p*_), after which they can either become symptomatic (*I*_ms_ and *I*_ss_) or remain without symptoms (*I*_*a*_). Symptomatic individuals can develop *mild* symptoms (*I*_ms_) or severe symptoms (*I*_ss_). When symptoms are severe, they are hospitalized (*H*). The final outcome of infected individuals is either recovery (*R*) or death (*D*)

We use an extended *SEIR* stochastic compartmental model (Fig. 1) in which we distinguish pre-symptomatic (*I*_*p*_), asymptomatic (*I*_*a*_), and symptomatic (*I*_ms_ and *I*_ss_) transmission by assuming different transmission rates, governed by different contact patterns during daytime and night-time as two time steps per simulated day. In particular, we assume that symptomatic individuals (both *mildly* symptomatic *I*_ms_ and *severely* symptomatic *I*_ss_) reduce their number of contacts (following observations made during the 2009 Influenza pandemic [20]) and their commute (school/work) mobility. A fraction of symptomatic adults can show severe symptoms and thereforef require hospitalization (*H*) [5, 21]. Once this happens, we assume that they cannot further infect other people due to isolation measures [22]. We assume that children have a 50% lower susceptibility to infection compared to adults [4, 23, 24]. Table 1 shows a summary of the model parameters and the distributional assumptions thereabout.

**Table 1:** Overview of the model parameters

Quantity	Median (95% CI)	Distribution	Source
Latent period (*ε*)	1.4 days ([0:7] days)	Exponential	[21, 60]
Pre-symptomatic period (*θ*)	2.4 days ([0:13] days)	Exponential	[21, 60]
Children infectivity (wrt adults)	0.5	—	[4, 23, 28, 61]
Proportion asymptomatic (*p*_*a*_)	0.5	—	[28, 61–63]
Proportion mild symptoms (*p*_*m*_)	0.5/0.476 (children/adults)	—	[23, 28, 61, 62]
Proportion severe symptoms (*p*_*s*_)	0 /0.024 (children/adults)	—	[9]
Symptomatic/asymptomatic period (*μ*)	2.4 days ([0:13] days)	Exponential	[53]
Symptom onset to hospitalization (*σ*)	4.7 days ([0:17] days)	Weibull	[53]
Hospital admission to death (*ξ*)	4 days ([1:9] days)	Log-logistic	[53]
Hospital admission to recovery (*τ*)	5 days ([1:10] days)	Weibull	[53]
Fitted parameter	Point estimate	(95% CI)	
Per-contact			
transmission probability (*β*)	0.0449	[0.0446:0.0451]	
Number of initial infected	14480	[12750:16217]	
Lockdown reduction in number of contacts	85%	[81%:89%]	
Time to reach full compliance	7 days	[7:7] days	

### Population mixing

According to the so-called *social contact hypothesis* [25] the number of contacts is proportional to the transmission probability of the disease, allowing to use empirically collected contact matrices to model disease transmission. Population mixing is informed by social contact data for different locations (work, home, school, transportation, leisure activity and other) during weekdays and weekends [15, 16], accessible through the Socrates tool [13, 14]. An asymptomatic individual interacts according to a contact matrix that is the sum of the contact matrices that correspond to different locations:

1 $$ \begin{aligned} C_{\text{asympt}}&=C_{\text{home}}+C_{\text{work}}+C_{\text{school}}+ C_{\text{leisure}}\\&\quad+ C_{\text{transport}}+ C_{\text{other}} \end{aligned}  $$

The locations considered are the main ones used to classify contact location in social contact surveys [26, 27] and include an overall category for leisure activities (e.g. going to the gym, to a bar, etc.). Contributions from work, school and transport contacts are considered only during daytime timesteps of the simulation, whereas other contributions are considered both for daytime and night-time timesteps. Given the strong age-specific severity of COVID-19, we assume that, when symptomatic, adults reduce their contacts in a location-specific fashion, as reported during the 2009 H1N1 pandemic [20]:

2 $$ \begin{aligned} C_{\text{sympt}}&=C_{\text{home}}+0.09\cdot C_{\text{work}}+0.06\cdot C_{\text{leisure}}\\&\quad+0.13\cdot C_{\text{transport}}+ 0.25\cdot C_{\text{other}} \end{aligned}  $$

We assume that children do not change behavior when symptomatic, as they are more likely to present fewer and milder symptoms as compared to adults [28–30].

When intervention measures are implemented (see “Exit strategies” section), location-specific contacts are reduced. This has an impact on both *C*_sympt_ and *C*_asympt_, implicitly assuming that a reduction in contacts because of symptoms is the same during the pre-pandemic and intervention period. The contact matrices then become:

3 $$ \begin{aligned} C_{\text{asympt}}&=C_{\text{home}}+p_{\mathrm{w}}\cdot C_{\text{work}}+p_{\mathrm{s}}\cdot C_{\text{school}}\\&\quad+p_{\mathrm{o}}\cdot C_{\text{leisure}}+p_{\mathrm{w}}\cdot C_{\text{transport}}+p_{\mathrm{o}}\cdot C_{\text{other}} \end{aligned}  $$

4 $$ \begin{aligned} C_{\text{sympt}}\!&=\!C_{\text{home}}+p_{\mathrm{w}}\cdot 0.09\cdot C_{\text{work}}+ p_{\mathrm{o}}\cdot0.06\cdot C_{\text{leisure}}\\&\quad+p_{\mathrm{w}}\cdot 0.13\cdot C_{\text{transport}}+ p_{\mathrm{o}}\cdot0.25\cdot C_{\text{other}} \end{aligned}  $$

where *p*_w_,*p*_s_,*p*_o_ are the percentages of contacts at work, at school and during leisure/other activities.

Contact matrices depend explicitly on the day of the week, as contact patterns during weekdays are profoundly different from contact patterns during the weekend.

### Population mobility

Data from the Belgian census [18] is used to infer the daily commuting network among different Belgian municipalities. These mobility patterns capture the regular, day-to-day movement of individuals to reach their working/studying place. Commuting individuals make contacts in their residence municipality during the night and in their work/school municipality during the day. This is captured via the force of infection, described in detail in the next section.

More details on population mobility can be found in the Supporting Information. We assume that telework and school closure, in addition to reducing contacts at work and at school, reduce the mobility of the corresponding age class. So, for example, if teleworking is reducing contact at work by 60%, also the mobility of adults is reduced by 60%. Considered values of mobility reduction for adults (*m*_*a*_) and children (*m*_*c*_) for each scenario are reported in Table 2.

**Table 2:** Timing and concepts of lockdown relief

	Timing start/end	Work & transportation contacts (%) (*p*_*w*_)	School contacts (%) (*p*_*s*_)	Mobility adults (%)(*m*_*a*_)	Mobility children(%) (*m*_*c*_)	Leisure & other contacts (%)(*p*_*o*_)
Phase 1 (work)	04-05/17-05	**20 [10-40]**	0	**20 [10-40]**	0	10
Phase 2 (school)	18-05/07-06	20 [10-40]	**20 [10-40]**	20 [10-40]	**20 [10-40]**	10
Phase 3 (leisure)	08-06/30-06	20 [10-40]	20 [10-40]	20 [10-40]	20 [10-40]	**20 [10-40]**
Summer holidays	01-07/31-08	20 [10-40]	**0**	20 [10-40]	**0**	20 [10-40]

Each phase is implemented incrementally with respect to the previous ones. Bold values highlight the changes with respect to the previous phase (i.e. the previous row). Intermediate parameter values are reported, with the full range between squared brackets. See sections ‘Population mixing’ and ‘Population mobility’ for parameter definitions

### Force of infection

The force of infection for age class *i* and patch *p* is computed at each time step as:

5 $$\begin{array}{@{}rcl@{}}  \lambda (i,p,t)&=\beta \sum_{j} \left[ \text{Susc}^{i} C^{\text{asympt}}_{i,j}(t) \text{Inf}^{\ j} \frac{I^{p}_{p,\ j}(t)+I^{p}_{a,\ j}(t)}{N^{p}(t)} \right. \\ &\qquad\left.+ \text{Susc}^{i} C^{\text{sympt}}_{i,j}(t)\text{Inf}^{\ j} \frac{I^{p}_{\text{ms},\ j}(t)+I^{p}_{\text{ss},\ j}(t)}{N^{p}(t)}\right] \end{array} $$

where $I^{p}_{x\ j}(t)$ is the number of infectious individuals of infection class *x* belonging to age class *j* present in patch *p* at time *t* and *N*^*p*^(*t*) is the total patch population at time *t*. Equation (5) can account for different susceptibility (Susc^*i*^) and infectivity (Inf^*j*^) for age classes *i* and *j*. The contact matrices used depend on time because of week/weekend cycles and because of the intervention strategies implemented at any given time (see following sections). The force of infection presents an additional dependence on time, as depending on the time steps commuting individuals may contribute to the force of infection of their residence patch or of their destination patch. This contribution is considered both in the infected terms $I^{p}_{x\ j}(t)$ as well as in the population term *N*^*p*^(*t*).

### Interventions implemented on the 14th of March 2020

Starting from Friday 13th of March at midnight, Belgian authorities have declared the nation-wide closure of schools and universities, together with restaurants, cafes and gyms. Also, public gatherings were banished. On the 17th of March, further dispositions were put in place, limiting mobility of people in addition to closing companies and shops offering non-essential services. We model interventions by reducing mixing and mobility in the population (see “Population mixing” and “Population mobility” sections), with a compliance that increases linearly with time and reaches full compliance on the 23rd of March.

### Calibration

To calibrate our model, we used publicly-available national data on daily hospital admissions [19]. We use Bayesian Optimization [31, 32] to maximize the likelihood of the simulated number of hospitalizations, given the observed data. We estimate the per-contact transmission probability (*β*), the number of initial infected, the reduction of the contact matrix during intervention with respect to the pre-pandemic period and time to reach full compliance (see Table 1). More details on the calibration procedure can be found in the Additional file 1.

### Exit strategies

The Belgian government lifted the lockdown gradually from the 4th of May onward. Table 2 shows a simplified summary of the different phases and their implementation. Changes with respect to the previous phase (i.e. the previous row) are shown in bold. In our scenario analysis we considered three phases:

- Phase 1: from the 4th of May, increasing the contacts made at work and during commuting by adults, to account for the increase of people going back to work. Mobility of adults increases accordingly.
- Phase 2: from the 18th of May, increasing contacts made at school and during commuting by children to account for school re-opening. Mobility of children increases accordingly.
- Phase 3: from the 8th of June, increasing contacts made during leisure and in other locations, to assess the impact of a possible re-opening of leisure activities.

For these phases we considered a compliance that increases linearly with time and reaches full compliance after one week.

### Case isolation

When extensive contact tracing and testing is available, a viable option for disease mitigation is to isolate individuals that are infected. We assume that case isolation affects both symptomatic and asymptomatic individuals and we present our results in terms of a synthetic quantity, the parameter *α*, that is the percentage of individuals entering the symptomatic/asymptomatic class (*I*_*a*_,*I*_ms_ and *I*_ss_) that are effectively isolated. We assume that these isolated individuals reduce their contacts by a factor of ten. We do not cover here how to link the target *α* to an optimal strategy for contact tracing and testing. Such a strategy should take into account feasibility thereof in terms of the number of index cases that can be traced, test features (e.g. sensitivity, specificity), and willingness to report contacts [22, 33, 34]. We also assume that no isolation of pre-symptomatic people is implemented (*I*_*p*_). We considered that case isolation can start at the beginning of phase 2 (i.e. on the 18th of May) or at the beginning of phase 3 (i.e. on the 8th of June), to assess the impact of delay in implementation.

## Results

### Impact of lockdown

**Fig. 2:**
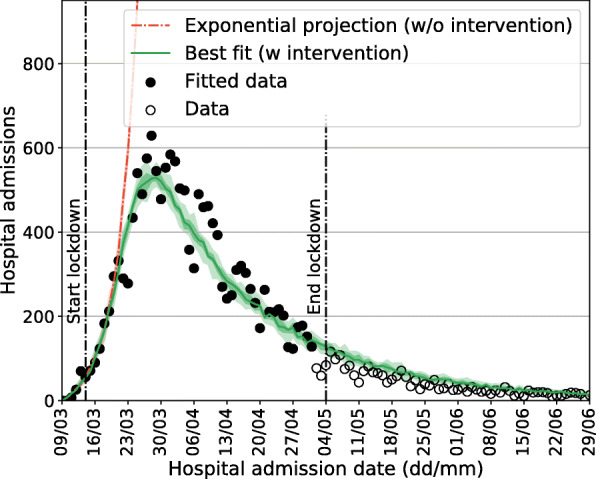
Model fitting. Data on hospital admissions is shown in comparison with the best-fit model. Black points are used to calibrate the model in the lockdown phase. In both panels median curves are shown along with 50% confidence intervals (CIs; dark shade) and 95% CI (light shade)

Figure 2 shows the daily number of new hospitalizations in the initial phase of the epidemic, compared with our best model fit. Hospitalization data up to the 21st of March are consistent with an exponential growth model with a doubling time of 3.09 days (95% CI [3.05:3.11]) (red line). Combined with our estimated model parameters, this results in a basic reproduction number *R*_0_=3.40 (95% CI [3.36:3.44]). A strong, periodic effect on the reported number of hospital admissions can be observed, most likely due to delays in hospitalization during weekends. The no-intervention model is in line with hospitalization data up to the 21th of March, showing that interventions took about one week to impact hospitalizations. The model including the effect of interventions (green line) is compatible with an overall reduction in the total number of contacts of 85% with respect to the period prior the COVID-19 pandemic (see Additional file 1 for additional information on contact matrices).

### Scenario analysis for lifting lockdown

**Fig. 3:**
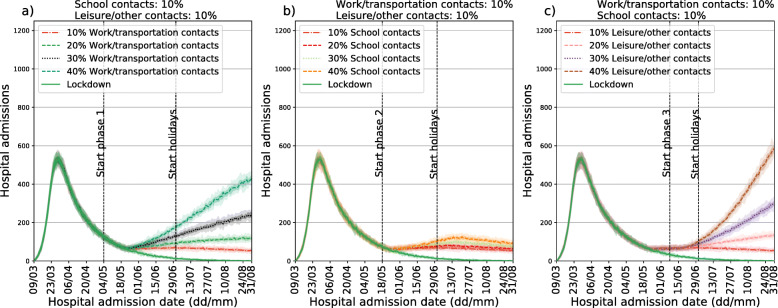
Exit scenarios using different timings and location-specific reductions. **a**: different implementations of phase 1 (work re-opening). **b**: different implementations of phase 2 (school re-opening). **c**: different implementations of phase 3 (leisure re-opening). The top of each panel shows the parameter values used. In all panels median curves are shown along with 50% confidence intervals (dark shade) and 95% CI (light shade). Color-code is consistent across panels, with the same color marking the same scenario in different panels

Figure 3 shows the impact of the different phases of the exit strategy on the number of new hospitalizations, considering different implementations (i.e. parameter values) for each phase. We present estimation of the number of new hospitalizations up to the 31st of August, as considering a longer timeframe would require additional assumptions with regard to social distancing after the summer period. Results for the whole year are reported in the Additional file 1 (Figure S4). In Fig. 3a, at the beginning of phase 1 (4th of May), contacts at work and on transportation are increased, ranging from 10% to 40% of pre-pandemic values. As expected, there is a delay between the implementation of the first phase and its effect on the number of hospital admissions: after 3 weeks the number of hospital admissions stops decreasing as compared to the lockdown scenario. One further week is required to see differences between the three implementations of phase 1. In Fig. 3b we show the impact of phase 2 (school re-opening) once phase 1 is implemented for the smallest value of contacts at work/transportation considered (10%). The percentage of school contacts ranges from 10% to 40%. In this case, the different curves start to diverge 4 weeks after the re-opening of schools. Summer school holidays, starting on the 1st of July have a considerable (delayed) effect on the number of hospital admissions only in the 40% school contacts scenario. In Fig. 3c we show the impact of phase 3, once phase 1 and 2 are implemented with the smallest values of the considered parameters. Different implementations (i.e. parameter values) of phase 3 give different results after three weeks. Comparing the three panels, it is clear that changing the implementation of phase 3 has a larger impact than changing implementation of phase 1 or 2. The larger impact of phase 3 (leisure/other activities) is confirmed when comparing all the scenarios we considered.

**Fig. 4:**
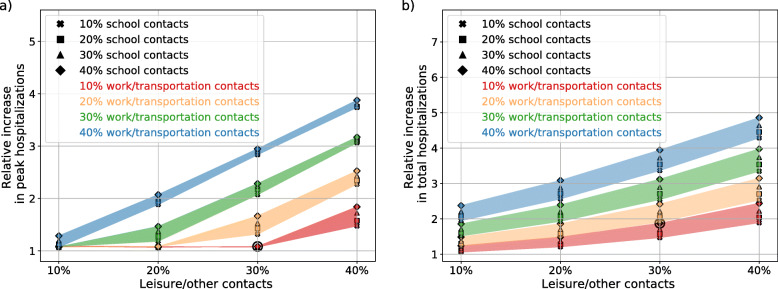
Summary of exit scenarios. **a**: peak value of daily hospital admissions up to the 31st of August. **b**: number of hospitalizations up to the 31st of August. In both panels the y-axis shows the relative variation with respect to the best-case (least contacts) scenario. A circle denotes the scenario used in the contact isolation analysis (Fig. 6)

Figure 4 shows the number of daily hospitalization and the cumulative number of hospitalizations up to the 31st of August (results up to the 31st of December are available in Additional file 1: Figure S4). Results are shown with respect to the scenario in which the lockdown would continue until the end of the simulation (green, solid line of panel a,b and c of Fig. 4) and that would result in 23.000 hospitalizations by the end of August. A smaller impact for school re-opening with respect to work and leisure re-opening is observed, both for peak hospitalizations and for total hospitalizations. Increasing the contacts at school by 10% (i.e. considering a different symbol marker but same color along the y-axis) has a smaller impact than increasing contacts at work (i.e. same symbol, different color along the y-axis) or leisure/other contacts (i.e. moving along the x-axis) of the same amount. Increasing contacts at work has a smaller impact in terms of peak hospitalizations than increasing leisure/other contacts; a similar impact is instead observed for the total number of hospitalizations. When considering results over the whole year (Additional file 1: Figure S4) the relative increase in the epidemic peak is weakly affected. The total final size, instead, increases for all scenarios, as the daily number of hospitalizations is summed up over a longer period of time.

**Fig. 5:**
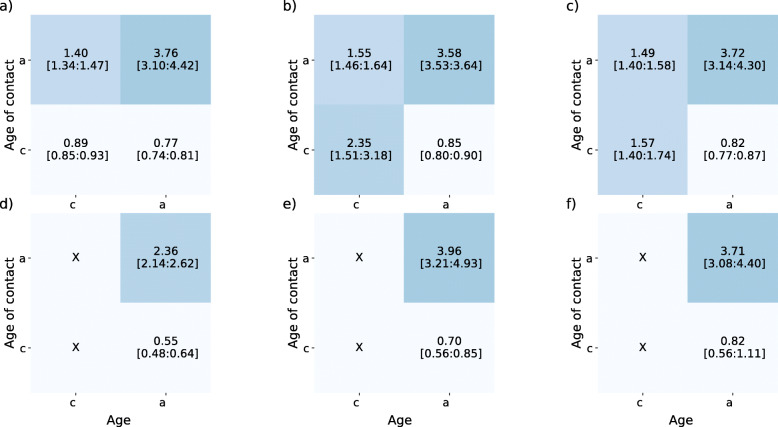
Comparison of model contact matrix and measured ones. **a**-**c**: Contact matrices for phase 1 (**a**), phase 2 (**b**) and phase 3 (**c**) in the simulated scenarios. For each matrix element we report the average value and the [min:max] interval over the different implementations of phases 1, phase 2 and phase 3 considered in Fig. 5. **d**-**f**: Contact matrices for phase 1 (**d**), phase 2 (**e**) and phase 3 (**f**) measured in a survey representative of the Belgian adult population. For each matrix element we report the average value and the 95% bootstrap confidence interval. Contacts of children participants, not measured in the survey, are marked with “X”. Data from [50], available at [59]

However, hospitalization data is compatible with the lockdown scenario (Fig. 2) up to the end of June. Comparison of the contact matrices used in the model with the results of a recent social contact survey targeting Belgian adults during and after the lockdown provides a means to interpret this. Figure 5 shows the measured contact matrices in comparison to the ones of the simulated scenarios. For the empirical contact matrices we report the average number of contacts, together with bootstrap confidence intervals (*n*=10,000), whereas for the ones of the model we report the average number of contacts and the min/max values considered in the scenarios listed in Table 2 and shown in Fig. 3. Our model uses a higher number of contacts during phase 1 with respect to empirical data, whereas for phase 2 and 3 we observe overlapping intervals for the number of contacts used in the model and measured by the empirical data. Data for children is however not available (hence marked with an “X” in Fig. 5), making a full comparison with empirical data not possible.

### Case isolation

**Fig. 6:**
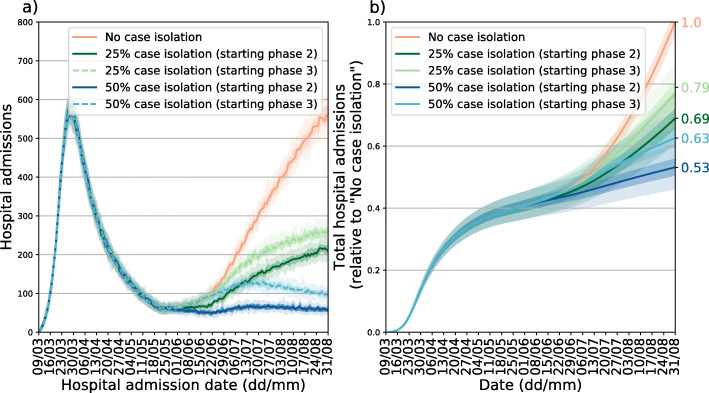
Effect of case isolation in a specific scenario. **a**: new hospitalizations per day. **b**: cumulative number of hospitalizations relative to the no case isolation scenario. All curves are obtained considering 40% of working contacts, 40% of contacts at school and 40% of leisure/other contacts with respect to pre-pandemic period (scenario denoted by a black circle in Fig. 4). In both panels median curves are shown along with 50% confidence intervals (dark shade) and 95% CI (light shade)

Figure 6 shows the impact of case isolation on the scenario marked with a circle in Fig. 4 (10% contacts at work/transportation, 40% contacts at school and 30% leisure/other contacts scenario marked with a circle in Fig. 4). The ability to isolate newly infected individuals has a considerable impact on the number of hospital admissions. The isolation of 25% of new cases is able to reduce the expected number of hospital admission at the end of August by 25%. The isolation of twice as many cases (50% instead of 25%) would lead to a reduction of 37% of admissions. Starting case isolation 3 weeks after (at the start of phase 3 instead of phase 2) lessens the reduction to 21% from 25%. A stronger effect of this delay is measured in the 50% case isolation scenario: in this case, starting the isolation at the start of phase 3 decreases the reduction in admissions from 37% to 28%.

## Discussion

We used a stochastic, discrete time, data-driven meta-population model to evaluate several scenarios for lifting the lockdown in three phases. The model includes data on pre-pandemic mobility and mixing, and is calibrated on hospital admissions. The initial phase of the COVID-19 epidemic in Belgium is characterized by a fast spread of the disease, with a doubling time of 3.09 days (95% CI [3.05:3.14]), in line with values from other countries [2, 5, 35–37]. Combined with our parameter choices, this results in *R*_0_=3.40 (95% CI [3.36:3.44]), which lies within the interval estimated in recent meta-analysis (mean =2.6, standard deviation =0.54 [38] and mean =3.28 [37]). Our model appropriately describes hospital admissions during the lockdown period if a strong reduction (85%) in the number of contacts is established. In this situation the number of hospital admissions starts to decrease 3 weeks after the start of the lockdown allowing the healthcare system to cope with ICU demands. At the end of the lockdown, the reproduction number is estimated to be 0.73 (95% CI [0.70:0.76]). Such a strong reduction in the average number of contacts marks the disruption that a lockdown has on everyday life. Studies in Wuhan and Shanghai [4] found an even stronger reduction in the number of contacts during lockdown, while a recent survey in the UK [38] measured a reduction of 75%. Preliminary analysis of social contact data collected in Belgium after the lockdown shows similar results as compared to [4] and [38], in line with our modelling results. Adherence to country-specific contact data is paramount, as intervention measures can vary substantially between countries, both in terms of implementation and in terms of compliance. Collecting country specific contact data during the different stages of the epidemic (i.e. before, during and after intervention) is therefore of crucial importance to adequately assess the impact of social distancing. Nevertheless, our knowledge of contact patterns before the COVID-19 crisis can be used to identify the relative impact of introducing social distancing in different locations. As we expect different locations to contribute to COVID-19 diffusion according to their location specific contact patterns, we can assess the impact of intervention strategies formalizing them in location specific reductions of contacts. In the current analysis this approach was taken, whilst considering a plausible range of reductions in social contacts in different circumstances. According to our model, leisure activities have the largest potential impact on the epidemic profile. This is consistent with leisure/other contacts accounting for 25% to 40% of the total contacts people make, according to representative surveys [26, 27]. However, the absence of a resurgence of hospitalizations by the end of June suggests that there is a smaller per-contact probability of transmission after lockdown with respect to pre-lockdown. This could be due to behavioral changes in how contacts are established (i.e. increased inter-personal distance or the wearing of face masks [39]) after the lockdown or to environmental factors (e.g. humidity and temperature [40]) that could affect transmission. As a matter of fact, surveys in Belgium [41] have documented a marked increase in outdoor contacts and face-mask wearing during the three phases of lockdown relief (Figure S3 in Additional file 1), supporting this hypothesis. In the light of that, our result are useful in establishing a hierarchy of location-specific contacts, but a careful interpretation of the absolute number of infections is necessary.

We observed less impact of school closure on hospital admissions in contrast to social mixing at work and during transport or leisure activities. First, as expected, school closure leads to observable effects only in those scenarios in which a consistent fraction (i.e. 40% or more) of school contacts are established in the population. Second, as children have a much lower probability of being symptomatic (and as such of being hospitalized) with respect to adults [37], increased diffusion among children increases the observed hospital admissions mostly indirectly, through the increase of infected adults. We tested, as a sensitivity analysis, a scenario in which children have the same susceptibility to the disease: in this case school closure would have a larger impact on the number of infections, especially in the children’s age class. The role of children is still unclear and, although their secondary attack rate in household is similar to the one of adults [29], there is evidence that they present smaller viral load [42–45] and reduced transmissibility [28, 46], together with a lower number of confirmed cases with respect to adults [23]. This increased susceptibility scenario is therefore unlikely, given the information on COVID-19 we have so far.

Since the expected resurgence in the number of hospitalizations is not observed, this suggests that the proportionality factor between conversational contacts and transmission rates postulated in the so-called social contact hypothesis [25] has changed from the lockdown to the post-lockdown period. This is likely due to behavioral changes (increased hygiene, prominence of outdoor over indoor community contacts, face-mask wearing, etc.) reducing the per-average contact transmission probability. For instance, surveys [41] in Belgium have documented a marked increase in outdoor contacts and face-mask wearing during the three phases of lockdown relief (Figure S3 in Additional file 1), supporting this hypothesis. In our results, isolation of newly infected individuals has an important impact on epidemic mitigation. Implementing case isolation would allow to re-establish social interactions while still ensuring epidemic containment. We stress here that although we quantified the reduction of spreading potential in terms of number of contacts, this may also come as a combination of different effects, for example when antivirals to be used in the early phase of the infection will become available [47]. Also, a fast setup is crucial: a 3 weeks delay in implementing case isolation leads to a considerable impact on the number of new hospital admissions. As a fast and reliable contact tracing is of foremost importance, several digital solutions have been proposed to match the need for personal information with privacy concerns [48, 49].

Our model assumptions result in a set of limitations. First, considering only two age classes does not allow to fully capture the heterogeneity involved in COVID-19 transmission, like increased burden on senior population. Including more age classes, however, would require assumptions for those age-specific parameters that have not been estimated for Belgium. In this sense our simplifying assumption, although less flexible, presents an easier to interpret picture.

The change in behavior for symptomatic individuals, and the corresponding reduction in the number of contacts, is informed by data collected during the 2009 H1N1 pandemic in the UK [20]. Although the COVID-19 pandemic presents different features with respect to the 2009 pandemic, a similar change of behavior in symptomatic individuals is expected. In particular, data collected in Belgium during and after the lockdown [50] has found that self-isolation when symptomatic is regarded as highly effective. Finally, although our model is specified at the municipality level, hospitalization data at the municipality level was not available at the time of conceiving of this study. We plan to expand our analysis at a smaller geographical scale in the future, to fully address heterogeneity in spatial transmission.

Other models have been applied to the emergence of COVID-19 in Belgium, either specifically [51–55] or in multi-country applications [56]. Using different model paradigms allows to focus on distinct aspects of the outbreak, like delay distributions of the clinical history of patients [53], a more detailed and age-specific handling of serological data with MCMC [52] or exploring individual-specific heterogeneities in transmissions and contact tracing options [51]. When evaluating intervention strategies with profound societal impact, ideally different models should be compared [57, 58].

## Conclusion

In conclusion, we show the predicted impact of a phase-based relief of lockdown measures taken in Belgium. Through validation using empirical data on social contacts and the observed trajectory of the epidemic, our results suggest that the per-contact probability of infection has changed from pre- to post-lockdown. While economic and societal needs urge governments to relieve strict distancing measures and mobility restrictions, caution is required. Contacts during leisure activities were found to be most influential, followed by professional contacts and school contacts, respectively, for an impending second wave of COVID-19. Regular re-assessment is crucial to adjust to evolving behavioral changes that can affect epidemic diffusion. In addition to social distancing, sufficient capacity for extensive testing and contact tracing is essential for successful mitigation.

## Supplementary Information

**Additional file 1** Supporting information.

## Data Availability

Demographic data is publicly available from Belgian Statistics. Surveillance data is publicly available and provided by the Belgian Scientific Institute for Public Health, Sciensano. Contact data are publicly available. Declarations
