# Supplementary material for: A data-driven metapopulation model for the Belgian COVID-19 epidemic: assessing the impact of lockdown and exit strategies
Source: BMC Infect Dis. 2021 May 30;21:503. doi: 10.1186/s12879-021-06092-w (PMC8164894; doi:10.1186/s12879-021-06092-w)
Supplement: Supplementary file 1 — Additional file 1 Supporting information. [file 12879_2021_6092_MOESM1_ESM.pdf]

SUPPORTING INFORMATION
# A data-driven metapopulation model for the Belgian COVID-19 epidemic: assessing the impact of lockdown and exit strategies

Pietro Coletti[1*], Pieter Libin[1,2,3], Oana Petrof[1], Lander Willem[4], Steven Abrams[1,5], Sereina A. Herzog[4,6], Christel Faes[1], James Wambua[1], Elise Kuylen[4], Philippe Beutels[4,7], Niel Hens[1,4]

April 9, 2021

[1] Data Science Institute, I-BioStat, UHasselt, Hasselt, Belgium. [2] Artificial Intelligence Lab, Department of computer science, Vrije Universiteit Brussel, Brussels, Belgium. [3] KU Leuven – University of Leuven, Department of Microbiology and Immunology, Rega Institute for Medical Research, Clinical and Epidemiological Virology, Leuven, Belgium. [4] Centre for Health Economic Research and Modelling Infectious Diseases, Vaccine & Infectious Disease Institute, University of Antwerp, Antwerp, Belgium. [5] Global Health Institute, Department of Epidemiology and Social Medicine, University of Antwerp, Antwerp, Belgium. [6] Institute for Medical Informatics, Statistics and Documentation, Medical University of Graz, Graz, Austria. [7] School of Public Health and Community Medicine, The University of New South Wales, Sydney, Australia.
* Corresponding author: pietro.coletti@uhasselt.be

## 1 Model details

### 1.1 Mobility model

Each municipality constitutes a patch in the model. Commuting data from the Belgian census [1] is used to construct the daily commuting network among different Belgian municipalities. These mobility fluxes correspond to people with daily mobility patterns between two municipalities, identified as their residence municipality and the municipality of their work/school. No data detailing mobility for other activities (e.g. leisure) is included.
We divide our simulational steps into daytime (even) and night-time (odd). At the beginning of every daytime time step, the number of individuals commuting from patch $i$ to other patches $T_i^a$ is generated via a time homogeneous Poisson process

$$T_i^a \sim \text{Poisson}(p_i^a, N_i) \tag{1}$$

where $N_i$ is the population of patch $i$ and $p_i^a$ is the probability that an individual in age class $a$ that resides in patch $i$ commutes to other patches (i.e. the ratio of the age specific commuting population of patch $i$ divided by the age specific population of the patch). After the number of commuters leaving the patch is computed, the number of commuters between any two patches $i$ and $j$ is modelled via a multinomial process:

$$T_{ij}^a \sim \text{Multinomial}(\hat{p}_{i,j}^a, T_i^a) \tag{2}$$

where $\hat{p}_{i,j}^a$ is the probability that an individual in age class $a$ resident in patch $i$ commutes to patch $j$ on a daily basis (i.e. the number of commuters between $i$ and $j$ divided by the full commuting population of patch $i$).

The probabilities of the two stochastic processes are computed from the 2001 Belgian Census, the last one providing commuting information (residence and working/school municipality) for the full Belgian population. The patch population ($N_i$, above) is given by population estimates for 2019. The percentages of individuals working on Saturdays and Sundays is used as a mobility reduction factor during weekend days.
At the beginning of every night-time time step, commuters move back to their residence location. Within country air travel was not considered due to negligible traffic.

## 1.2 Compartmental model

In every patch of the metapopulation system, a two-age class SEIR model is defined [2]:

$$\frac{dS}{dt} = -\beta S \frac{C(t)}{N} I \quad (3)$$

$$\frac{dE}{dt} = \beta S \frac{C(t)}{N} I - \epsilon E \quad (4)$$

$$\frac{dI}{dt} = \epsilon E - \mu I \quad (5)$$

$$\frac{dR}{dt} = \mu I \quad (6)$$

$$(7)$$

where $S$,$E$,$I$,$R$, are the 2-component vectors (e.g. $S = \begin{pmatrix} S_1 \\ S_2 \end{pmatrix}$), whose elements are the number of individuals in the two age classes at time $t$ in the susceptible, exposed, infected and recovered compartment. Here $N$ is the considered population and $C(t)$ is the contact matrix, the 2x2 matrix defining age class mixing. The parameter $\beta$ is the per-contact infection probability, whereas $\epsilon$ and $\mu$ correspond to the latent period and the recovery time.
We implemented the $SEIR$ equations defining transition probabilities for small $dt$ and age class $i$ as:

$$p_i^{S\to E}(t) = 1 - e^{-\beta\lambda(i,t)dt} \quad (8)$$

$$p_i^{E\to I} = 1 - e^{-\epsilon dt} \quad (9)$$

$$p_i^{I\to R} = 1 - e^{-\mu dt} \quad (10)$$

$$(11)$$

where we have inserted the force of infection (FOI) for age class $i$ in patch $p$, $\lambda(i,t)$. These transition probabilities are defined in each patch and, while $p_i^{E\to I}$ and $p_i^{E\to I}$ are the same for all patches, the force of infection depends both on time and the patch considered. The expression for the FOI for age class $i$ in patch $p$ is (same as main paper):

$$\lambda(i,p,t) = \beta \sum_j \Big[ \quad \text{Susc}^i C_{i,j}^{\text{asympt}}(t) \text{Inf}^j \frac{I^p_{p,\ j}(t) + I^p_{a,\ j}(t)}{N^p(t)} + \quad (12)$$

$$+ \quad \text{Susc}^i C_{i,j}^{\text{sympt}}(t) \text{Inf}^j \frac{I^p_{\text{ms},\ j}(t) + I^p_{\text{ss},\ j}(t)}{N^p(t)} \Big] \quad (13)$$

where $I^p_{x,\ j}(t)$ is the number of infectious individuals of infection class $x$ ()belonging to age class $j$ present in patch $p$ at time $t$ and $N^p(t)$ is the total patch population at time $t$. Equation (12) can account for different susceptibility ($\text{Susc}^i$) and infectivity ($\text{Inf}^j$) for each age classes $i$ and $j$. The contact matrices used depend on time because of week/weekend cycles and because of the intervention strategies implemented at any given time (see section "Population mixing" of the main

paper). As mentioned, we split our time steps into daytime time steps (even) and night-time time steps (odd). During even time steps, commuters contribute to the force of infection of their destination patch. During odd time steps, they contribute to the force of infection of their origin patch. This contribution is considered both in the $I^p_{x,\ j}(t)$ terms as well as in the population term $N^p(t)$.
From the transition probabilities, the corresponding number of new individuals of age class $i$ in the compartments is generated using binomial processes:

$$
\begin{aligned}
E_{\text{new},i} &\sim \text{Binomial}(p_i^{S\to E}(t), S_i(t)) && (14)\\
I_{\text{new},i} &\sim \text{Binomial}(p_i^{E\to I}(t), E_i(t)) && (15)\\
R_{\text{new},i} &\sim \text{Binomial}(p_i^{I\to R}(t), I_i(t)) && (16)\\
& && (17)
\end{aligned}
$$

Commuters are modeled with separate compartments, in order to track them in their movements from residence to destination and back.

## 2 Software details

The code is developed in C++ and makes use of the boost libraries [3]. One run for the whole population of Belgium takes less than three minutes on a regular laptop (Intel® Core™ i7-6700HQ CPU @ 2.60GHz). All results presented in this manuscript are generated on the tier-2 infrastructure of the VSC-cluster [4] using one of the Skylake nodes, each equipped with 2 Xeon Gold 6140 CPUs@2.3 GHz (Skylake) (18 cores each). For each scenario we considered 30 different stochastic realizations, presenting results in terms of median value and percentile intervals. Data analysis is performed in R and Python. The Bayesian optimization is implemented through the Python package pyGPGO [5].

## 3 Model calibration

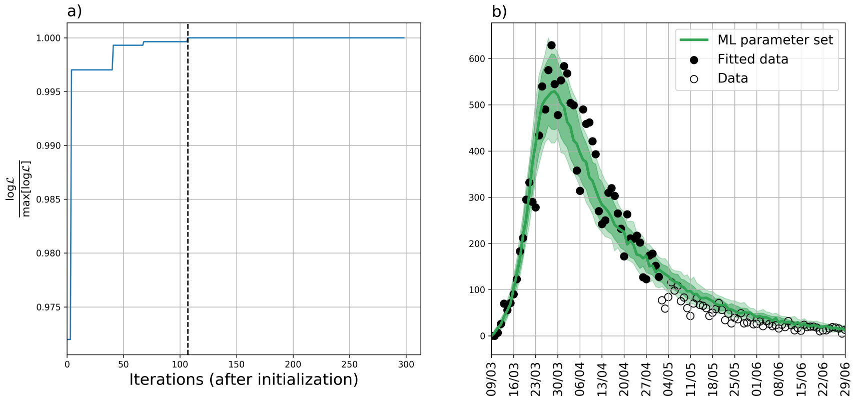


**Figure 1: Calibration results.** (**a**): Maximum of log-likelihood as a function of the iteration number. A vertical dashed line marks the last improvement in log-likelihood. (**b**): Simulated number of hospitalizations over time for the Maximum Likelikihood (ML) parameter set (line) and 95% confidence interval (shaded area) considering min (max) values of all parameters.

We calibrated our model to national data on hospital admission [6] by maximizing the likelihood with respect to hospitalization counts using Bayesian optimization [7] .

To estimate the probability of developing severe symptoms ($p_s$ in Figure 1 of the main paper) we compared results from a sero-prevalence [8] survey with hospitalization data [6]. We estimated this probability as the ratio of the total number of hospitalizations ($N_h$) divided by the total number of cases ($N_c$). The first quantity is obtained by summing the number of new hospitalizations over time, while the second quantity can be inferred by multiplying the sero-prevalance by the total Belgian population.

$$p_s = \frac{N_h}{N_c} = \frac{\sum_{t_i=1}^{N_t} H(t_i)}{S(N_t - t^*) \cdot N} \tag{18}$$

In the above, $H(t_i)$ is the number of new hospitalizations at day/time $t_i$, $N_t$ is the number of times/days considered, $N$ denotes the total Belgian population and $t^*$ takes into account the time needed to build up antibodies against SARS-CoV-2 that can be detected in the sero-prevalence survey. In our calculation we used a $t^*$ equal to 7 days and considering the midpoint of the week as the data-collection day of the sero-prevalence survey. We evaluated eq. (18) using the latest date of serological data collection (11th of May) not affected by re-opening after lockdown. This resulted in a $p_s$ of 0.024 (CI [0.020 : 0.025]).

Our model takes parameters: the probability of per-contact transmission, the number of initial infected on the 9th of March, the reduction in contact frequency during lockdown and time needed to reach full compliance in lockdown adherence (see Table 1 of main paper). We considered data up to the 4th of May and we maximised the likelihood, following the approach described below.

We considered hospitalization data to follow a Poisson distribution and we found our point estimates for the parameter maximising the log-likelihood ($\mathcal{LL}$):

$$\mathcal{LL} = \sum_{t_i} [-H(t_i) * \log \hat{H}(t_i) + \hat{H}(t_i)] \tag{19}$$

where $H(t_i)$ is the number of new hospitalization at day/time $t_i$, $\hat{H}(t_i)$ is the simulated number of new hospitalizations and the log-likelihood $\mathcal{LL}$ is written up to a constant factor. In order to fit the the parameters of our computationally intensive simulator to the likelihood, we use Bayesian optimization [7]. Our Bayesian optimization procedure uses a Matèrn kernel [9] and an acquisition function that samples parameters according to the expected improvement algorithm [7]. To achieve a fit, we obtained $\tilde{N}$ samples from our simulator (after 10 initialization steps) and assumed that the process converged if in the last $\frac{\tilde{N}}{2}$ iterations no improvement in the log likelihood was obtained. This resulted in a total of 300 sampled iteration (see Figure 1 a) ). To estimate the confidence interval of our parameter estimates, we evaluated numerically the Fischer information at the maximum of the likelihood [10].

Visual inspection of the fit shows a good agreement with the number of hospitalizations over time. Uncertainty of parameter estimates is affecting mostly the number of initial infected and the per-contact transmission probability. Time to reach full compliance is compatible with 7 days and larger/smaller values (e.g. 8 or 6 days) are outside the confidence interval. Table 1 of the main paper shows best parameters estimates and the corresponding confidence interval.

## 4 Contact matrices

Figure 2 shows the contact matrices for weekdays before lockdown ( panels a and b ) and during lockdown (panels c and d). A strong reduction in the total number of contacts during lockdown with respect to pre-lockdown ($\sim 85\%$) is obtained from the fitting procedure.

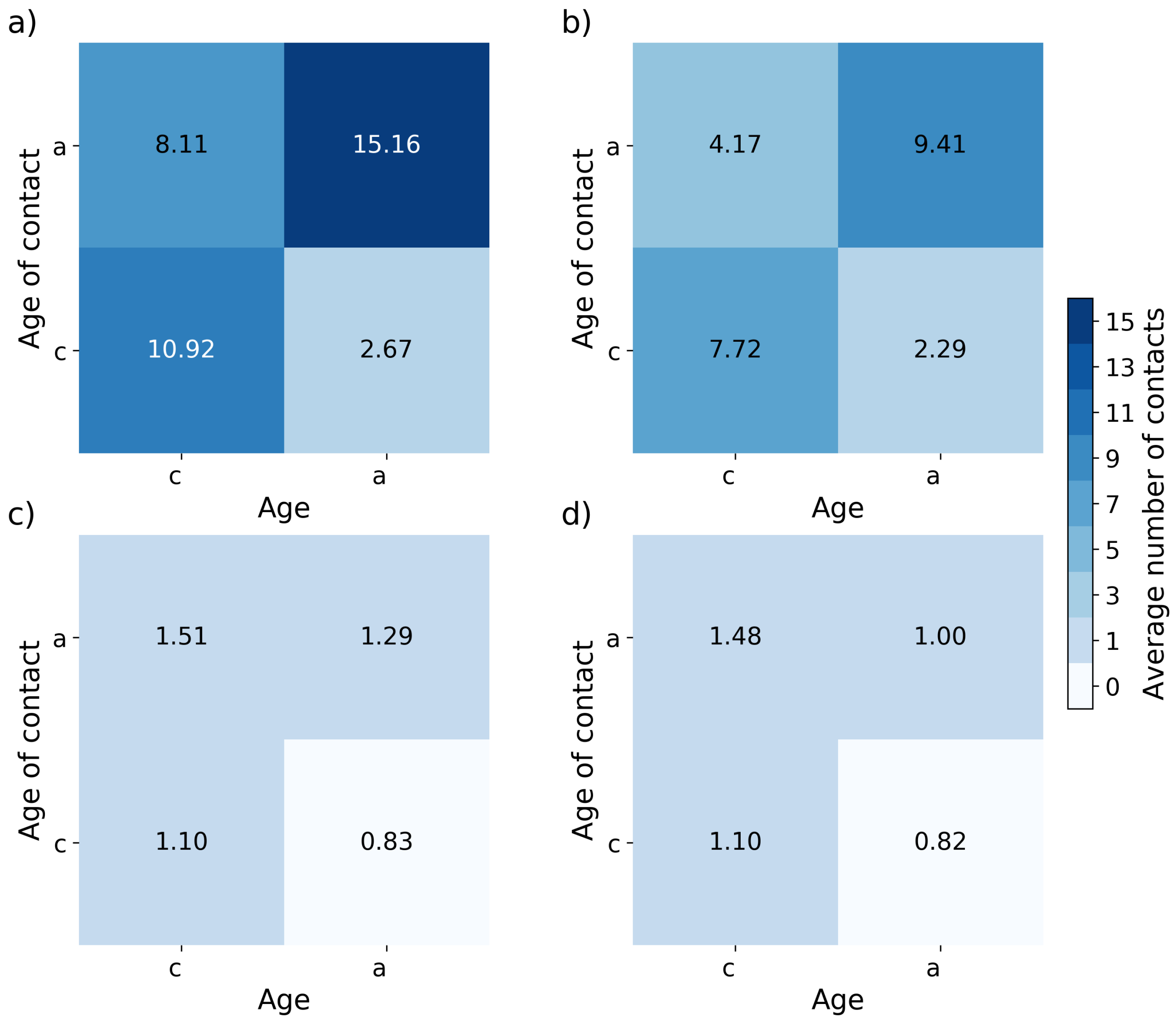


**Figure 2: Contact matrices for weekdays.** (**a**): Contact matrix for pre-symptomatic (compartment $I_p$) before lockdown. (**b**): Contact matrix for symptomatic/asymptomatic (compartments $I_a$,$I_{\mathrm{ms}}$,$I_{\mathrm{ss}}$) before lockdown. (**c**): Contact matrix for pre-symptomatic (compartment $I_p$) during lockdown. (**d**): Contact matrix for symptomatic/asymptomatic (compartments $I_a$,$I_{\mathrm{ms}}$,$I_{\mathrm{ss}}$) during lockdown.

## 5 Scenario with increased infectivity of children

We considered a scenario in which children are as infectious as adults (Figure 5). The increased role of children in disease spread impacts our predicted scenarios as following. First, school closure has a larger impact on the epidemic profile and in case of a large number of contacts at school (60% of pre-pandemic values) the epidemic is peaking earlier. Also, as expected, a larger impact of summer school closure is observed. In this scenario, school closure has a larger impact overall than re-opening working places, while it is still less relevant than re-opening leisure activities.

## References


[1] StatBel, the Belgian statistical office. https://statbel.fgov.be/en

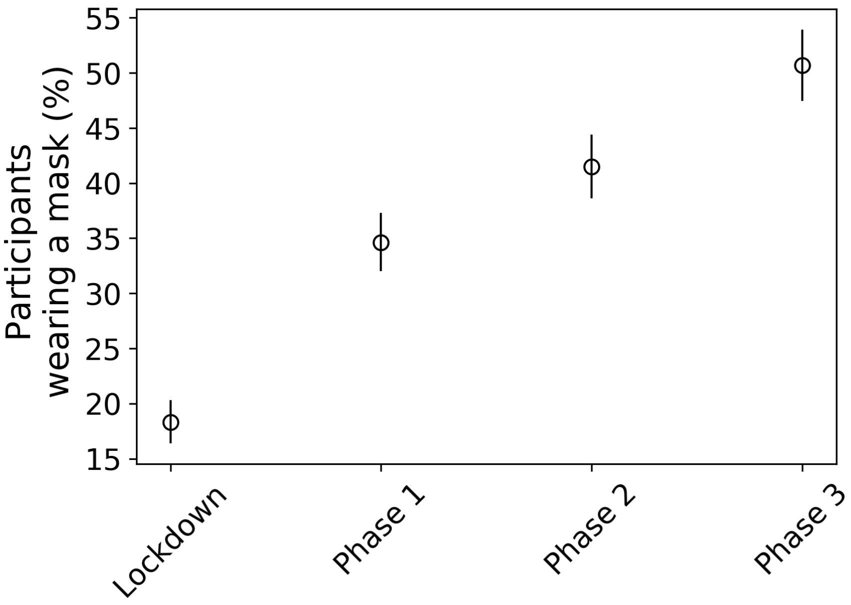


**Figure 3: Percentage of face-mask using among Belgian adult population.** Percentage of adults participating in the CoMix survey [11] that wore a mask.

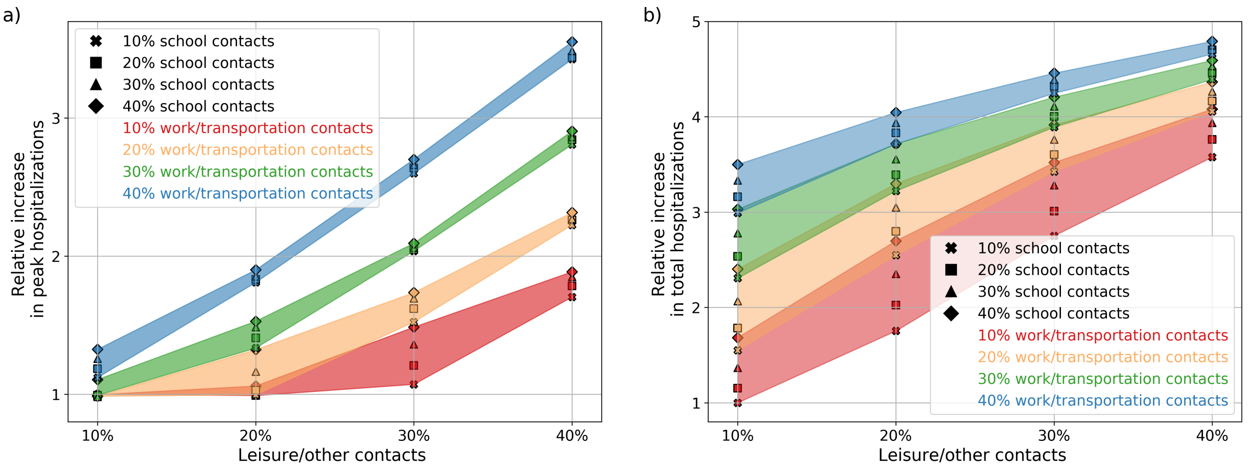


**Figure 4: Summary of exit scenarios (up to the 31st of December).**(**a**): peak value of daily hospital admissions for different scenarios up to the 31st of December. (**b**): number of hospitalizations up to the 31st of December. In both panels the y-axis shows the relative variation with respect to the best-case (least contacts) scenario.


[2] Keeling, M.J., Rohani, P.: Modeling Infectious Diseases in Humans and Animals. Princeton University Press, ??? (2008)

[3] Boost libraries. https://www.boost.org/

[4] Vlaams Supercomputer Centrum. https://www.vscentrum.be/

[5] Jiménez, J., Ginebra, J.: pygpgo: Bayesian optimization for python. Journal of Open Source Software **2**(19), 431 (2017). doi:10.21105/joss.00431

[6] Sciensano, Covid-19 Belgium Epidemiological Situation. https://datastudio.google.com/embed/u/0/reporting/c14a5cfc-cab7-4812-848c-0369173148ab/page/tpRKB

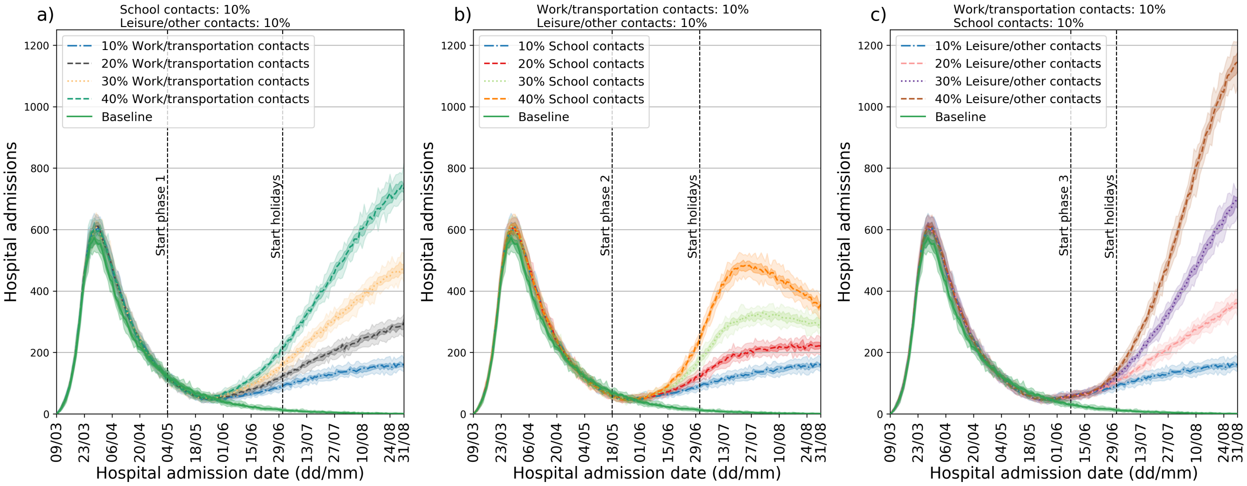


**Figure 5: Exit scenarios for scenario with equal susceptibility of children and adults.** (**a**): different implementations of phase 1 (work re-opening). (**b**): different implementations of phase 2 (school re-opening). (**c**): different implementations of phase 3 (leisure re-opening). The top of each panel shows the parameter values used. Color-code is consistent across panels, with the same color marking the same scenario in different panels.


[7] Shahriari, B., Swersky, K., Wang, Z., Adams, R.P., de Freitas, N.: Taking the human out of the loop: A review of bayesian optimization. Proceedings of the IEEE **104**(1), 148–175 (2016). doi:10.1109/JPROC.2015.2494218

[8] Herzog, S., De Bie, J., Abrams, S., Wouters, I., Ekinci, E., Patteet, L., Coppens, A., De Spiegeleer, S., Beutels, P., Van Damme, P., Hens, N., Theeten, H.: Seroprevalence of igg antibodies against sars coronavirus 2 in belgium: a prospective cross-sectional study of residual samples. medRxiv (2020). doi:10.1101/2020.06.08.20125179. https://www.medrxiv.org/content/early/2020/06/09/2020.06.08.20125179.full.pdf

[9] Genton, M.G.: Classes of kernels for machine learning: A statistics perspective. J. Mach. Learn. Res. **2**, 299–312 (2002)

[10] Fisher, R.A., Russell, E.J.: On the mathematical foundations of theoretical statistics. Philosophical Transactions of the Royal Society of London. Series A, Containing Papers of a Mathematical or Physical Character **222**(594-604), 309–368 (1922). doi:10.1098/rsta.1922.0009. https://royalsocietypublishing.org/doi/pdf/10.1098/rsta.1922.0009

[11] Coletti, P., Wambua, J., Gimma, A., Willem, L., Vercruysse, S., Vanhoutte, B., Jarvis, C.I., Van Zandvoort, K., Edmunds, J., Beutels, P., Hens, N.: Comix: comparing mixing patterns in the belgian population during and after lockdown. Scientific Reports **10**(1), 21885 (2020). doi:10.1038/s41598-020-78540-7
